# A comprehensive in silico analysis and experimental validation of miRNAs capable of discriminating between lung adenocarcinoma and squamous cell carcinoma

**DOI:** 10.3389/fgene.2024.1419099

**Published:** 2024-09-23

**Authors:** Zahra Javanmardifard, Saeid Rahmani, Hadi Bayat, Hanifeh Mirtavoos-Mahyari, Mostafa Ghanei, Seyed Javad Mowla

**Affiliations:** ^1^ Department of Molecular Genetics, Faculty of Biological Sciences, Tarbiat Modares University, Tehran, Iran; ^2^ School of Computer Science, Institute for Research in Fundamental Science (IPM), Tehran, Iran; ^3^ Biochemical Neuroendocrinology, Institut de Recherches Cliniques de Montréal (IRCM), affiliated to the Division of Experimental Medicine, Faculty of Medicine and Health Sciences, McGill University, Montreal, QC, Canada; ^4^ Lung Transplantation Research Center (LTRC), National Research Institute of Tuberculosis and Lung Diseases (NRITLD), Shahid Beheshti University of Medical Sciences, Tehran, Iran; ^5^ Chemical Injuries Research Center, Systems Biology and Poisonings Institute, Baqiyatallah University of Medical Sciences, Tehran, Iran

**Keywords:** microRNA, NSCLC, machine learning, feature selection, prognosis, qPCR

## Abstract

**Background:**

Accurate differentiation between lung adenocarcinoma (AC) and lung squamous cell carcinoma (SCC) is crucial owing to their distinct therapeutic approaches. MicroRNAs (miRNAs) exhibit variable expression across subtypes, making them promising biomarkers for discrimination. This study aimed to identify miRNAs with robust discriminatory potential between AC and SCC and elucidate their clinical significance.

**Methods:**

MiRNA expression profiles for AC and SCC patients were obtained from The Cancer Genome Atlas (TCGA) database. Differential expression analysis and supervised machine learning methods (Support Vector Machine, Decision trees and Naïve Bayes) were employed. Clinical significance was assessed through receiver operating characteristic (ROC) curve analysis, survival analysis, and correlation with clinicopathological features. Validation was conducted using reverse transcription quantitative polymerase chain reaction (RT-qPCR). Furthermore, signaling pathway and gene ontology enrichment analyses were conducted to unveil biological functions.

**Results:**

Five miRNAs (miR-205-3p, miR-205-5p, miR-944, miR-375 and miR-326) emerged as potential discriminative markers. The combination of miR-944 and miR-326 yielded an impressive area under the curve of 0.985. RT-qPCR validation confirmed their biomarker potential. miR-326 and miR-375 were identified as prognostic factors in AC, while miR-326 and miR-944 correlated significantly with survival outcomes in SCC. Additionally, exploration of signaling pathways implicated their involvement in key pathways including PI3K-Akt, MAPK, FoxO, and Ras.

**Conclusion:**

This study enhances our understanding of miRNAs as discriminative markers between AC and SCC, shedding light on their role as prognostic indicators and their association with clinicopathological characteristics. Moreover, it highlights their potential involvement in signaling pathways crucial in non-small cell lung cancer pathogenesis.

## 1 Introduction

As the second most frequently diagnosed cancer worldwide, lung cancer (LC) ranks as the deadliest among malignant tumors, contributing to one-fifth of total cancer deaths (Sung et al., 2021). LC is broadly classified into two main subtypes: non-small cell lung cancer (NSCLC) and small cell lung cancer (SCLC). NSCLCs make up approximately 80% of all lung cancer cases, with adenocarcinoma (AC) and squamous cell carcinoma (SCC) emerging as the two most prevalent subtypes, accounting for around 40% and 20% of lung cancers, respectively (Zheng, 2016).

The heterogeneity of NSCLC subtypes results in distinct therapeutic approaches for each specific histological type. Therefore, to individualize treatment strategies for each patient, it is crucial to establish a precise histological classification for NSCLC (Charkiewicz et al., 2016). While conventional morphological assessment of tissue sections persists as the established gold standard for diagnosing LC, it is accompanied by inherent challenges and limitations. Furthermore, immunohistochemistry techniques have provided only partial assistance in improving the accuracy of NSCLC subtyping, occasionally leading to potential difficulties in distinguishing between AC and SCC (Tane et al., 2014; Charkiewicz et al., 2016; Patnaik et al., 2015). Hence, there is a pressing requirement for identifying and validating biomarkers facilitating precise subclassification of NSCLC and ultimately contributing to enhanced clinical outcomes.

MicroRNAs (miRNAs), as a class of conserved small non-coding RNA molecules, are responsible for regulating the expression of genes involved in a vast variety of biological processes (Hill and Tran, 2021). The aberrant expression of miRNAs has been revealed to make a substantial contribution to the development and progression of various cancers including NSCLCs (Hill and Tran, 2021; Florczuk et al., 2017). Owing to the correlation between expression levels of different miRNAs and histological subtypes of NSCLC and high stability of these small non-protein-coding RNAs in formalin-fixed tissues and biological fluids, miRNAs can be considered to have potential biomarker value for differentiating between AC and SCC patients (Du et al., 2017; Landi et al., 2010; Mlcochova et al., 2014; Kosaka et al., 2010; Xi et al., 2007).

Over the past few years, several studies have been conducted to unveil the usefulness of miRNAs for determination of NSCLC subtypes. For instance, Hamamoto et al. proposed a combination of three miRNAs (miR-196b, miR-205 and miR-375) for the classification of AC and SCC subtypes (Hamamoto et al., 2013). Sun et al. demonstrated that, while miR-29b-3p was upregulated in AC, the higher expression of miR-105-5p was evident in SCC samples (Sun et al., 2017). All members of the let-7 family were also reported to be downregulated in SCC histology (Landi et al., 2010). Zhang et al. revealed that miR-205, miR-221, and miR-30e had lower expression in AC, whereas the expression levels of miR-29b, let-7e, and miR-125a-5p were shown to be higher in patients with AC compared with SCC (Zhang et al., 2012).

**FIGURE 1:**
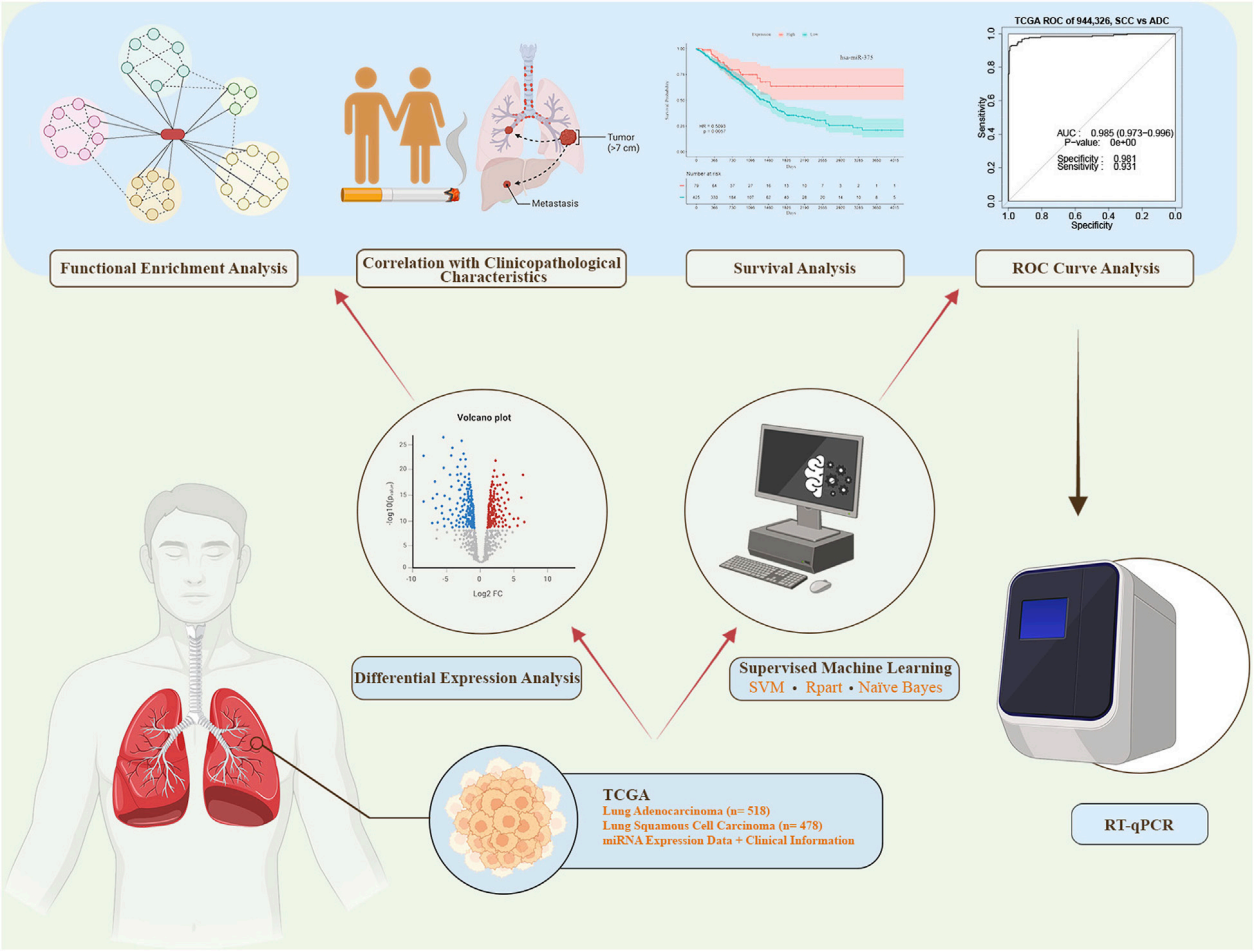
The workflow of this study. miRNA expression data and corresponding clinical information were obtained from The Cancer Genome Atlas (TCGA). Differential expression analysis and supervised machine learning using three distinct methods were conducted. The top-ranked miRNAs from both approaches were selected for further analyses, including receiver operating characteristic (ROC) curve analysis, survival analysis, correlation analysis, and functional enrichment analysis. miRNAs demonstrating high sensitivity and specificity in the ROC analysis were subsequently chosen for experimental validation via reverse transcription quantitative polymerase chain reaction (RT-qPCR). SVM: Support Vector Machine.

In this study, we conducted a comprehensive analysis by employing both differential expression analysis and supervised machine learning feature selection methods. This combined approach led to the identification of miRNAs demonstrating high discriminatory value for distinguishing between AC and SCC. Subsequently, we performed receiver operating characteristic (ROC) curve analysis to assess the diagnostic power and survival analysis to evaluate the prognostic value of the identified miRNAs. We also explored the correlation between miRNA expression levels and clinicopathological characteristics. Furthermore, we detected the expression levels of the two candidate miRNAs (miR-944 and miR-326) in formalin-fixed paraffin-embedded (FFPE) tissue samples from AC and SCC patients using reverse transcription quantitative polymerase chain reaction (RT-qPCR). Finally, we revealed the biological functions and signaling pathways associated with the identified miRNAs through functional enrichment analysis. The diagram illustrating the process of this study is presented in Figure 1.

## 2 Materials and methods

### 2.1 Data acquisition and identification of differentially expressed miRNAs

We obtained mature miRNA expression data and corresponding clinical information from the lung adenocarcinoma (LUAD) project, including 518 tumor and 49 adjacent normal samples, and lung squamous cell carcinoma (LUSC) project, including 478 tumor and 45 adjacent normal tissues, from The Cancer Genome Atlas (TCGA) data portal (https://portal.gdc.cancer.gov/). We utilized the TCGAbiolinks R package (RRID: SCR_017683) for data acquisition (Colaprico et al., 2016). To address potential inter-project library size biases, we performed count per million (CPM) normalization on the acquired datasets for further analysis.

The “limma” R package (RRID:SCR_010943) was then applied to compare the miRNA expressions between AC tumors and SCC group, identifying differentially expressed miRNAs (DEMs) (Ritchie et al., 2015). |log2FC|>2 and false discovery rate (FDR) adjusted *p*-value < 0.001 were considered as screening criteria for determining the DEMs.

### 2.2 Feature selection and classification

Three distinct supervised machine learning approaches, including Support Vector Machine (SVM), Decision trees and Naïve Bayes, were carried out to identify candidate miRNA biomarkers capable of distinguishing patients with AC from the SCC group. Different classifiers were used to enhance the expectation of generalization of biomarkers. Due to the structural differences among these classifiers, a set of informative miRNAs with acceptable performance across all of them has a greater chance of generalization. The “e1071” package was used to perform SVM, Naïve Bayes, and Rpart (Recursive Partitioning and Regression Trees) (Dimitriadou et al., 2006). The Robust Rank Aggregation (RRA) method was implemented using the “RobustRankAggreg” package (RRID:SCR_024299) in R to merge feature sets ranked by different methods based on their performance (Kolde et al., 2012).

### 2.3 ROC curve analysis

The ability of miRNAs to distinguish patients with AC from those with SCC was assessed by generating ROC curves and calculating the area under the curve (AUC) with a 95% confidence interval (CI). To construct the ROC curve, which detects the optimal threshold to handle the trade-off between specificity and sensitivity, a linear classifier in the original feature space is commonly used. In our study, SVM was utilized to enhance the efficiency and facilitate the handling of multiple features simultaneously. For ROC analysis, the “pROC” package (RRID:SCR_024286) was employed, and for SVM implementation, the “e1071” package was used (Robin et al., 2011). The input dataset was randomly split into two sets, with 3/5 for training and 2/5 for testing. To address the unbalanced class situation, the training set was resampled.

### 2.4 Correlation with clinical characteristics

To explore the association between miRNA expression and clinicopathological features, we classified labels into numerical and categorical variables. We used Pearson correlation for numerical variables, the nonparametric Wilcoxon rank-sum test (Mann–Whitney U test) for two-group categorical variables, and the nonparametric Kruskal–Wallis test for scenarios involving more than two groups.

### 2.5 Survival analysis

Survival curves, generated using Kaplan-Meier analysis, were plotted to assess the prognostic potential of miRNAs. To explore the correlation between miRNA expression and overall survival of patients, the Cox proportional hazards model was applied. Subsequently, the log-rank test was employed to evaluate the significance of differences in survival patterns. For plotting Kaplan-Meier curves and measuring the significance of hazard ratios between high-expression and low-expression groups, we utilized the “survival” (RRID:SCR_021137) and “survminer” (RRID:SCR_021094) packages in R (Therneau and Lumley, 2015; Kassambara et al., 2017).

### 2.6 Target genes identification and functional enrichment analysis

The experimentally validated target genes of the top 25 DEMs were obtained from miRTarBase using the multiMiR package in R (Ru et al., 2014). Next, Pearson correlation analysis was employed to assess the correlations between gene and miRNA expression levels. Genes showing significant negative correlation (*p*-value <0.05) were subjected to Kyoto Encyclopedia of Genes and Genomes (KEGG) pathway and gene ontology (GO) enrichment analysis using Enrichr (Xie et al., 2021; Kuleshov et al., 2016; Chen et al., 2013).

### 2.7 Clinical specimens

This study was conducted with the approval of the Research Ethics Committee of Tarbiat Modares University (ID: IR.MODARES.REC.1400.081). FFPE tissue samples from 50 NSCLC patients were obtained from the archives of Masih Daneshvari Hospital (Tehran, Iran). This study involved the collection of human tissue specimens without conducting any experiments on human subjects. Among these 50 FFPE samples, 29 were AC, and the remaining 21 were SCC. All specimens were examined and classified by pathologists according to the World Health Organization classification of lung tumors.

### 2.8 RNA extraction and RT-qPCR

To extract RNA, paraffin was first removed from four 10 µm FFPE tissue sections using Xylene. Subsequently, the dewaxed tissues were washed with absolute ethanol, followed by digestion with Proteinase K. After the deparaffinization process, total RNA isolation was carried out using TRIzol reagent (Thermo Fisher Scientific, USA). DNA contamination was then eliminated using Turbo DNase (Thermo Fisher Scientific, USA). The quality and quantity of purified RNAs were measured using NanoDrop (Thermo Fisher Scientific, USA). The purified RNAs were polyadenylated with *E.coli* poly (A) polymerase (New England Biolabs, USA), and the reverse transcription reaction was conducted using anchored oligo (dT) primers and AddScript cDNA Synthesis Kit (AddBio, Korea) to determine the expression levels of miR-944 and miR-326.

For amplification and quantification of miRNAs, a StepOne Plus System (Applied Biosystems, USA) was used with RealQ Plus 2x Master Mix Green, High ROX™ (Ampliqon, Denmark). Expression levels of miR-944 and miR-326 were normalized to that of U48, selected as an endogenous control, and their relative expressions were determined using the 2^−ΔΔCT^ method. The sequence of primers and oligonucleotides used in reverse transcription and qPCR are provided in Supplementary Table S1.

## 3 Results

### 3.1 Identification of DEMs

**FIGURE 2:**
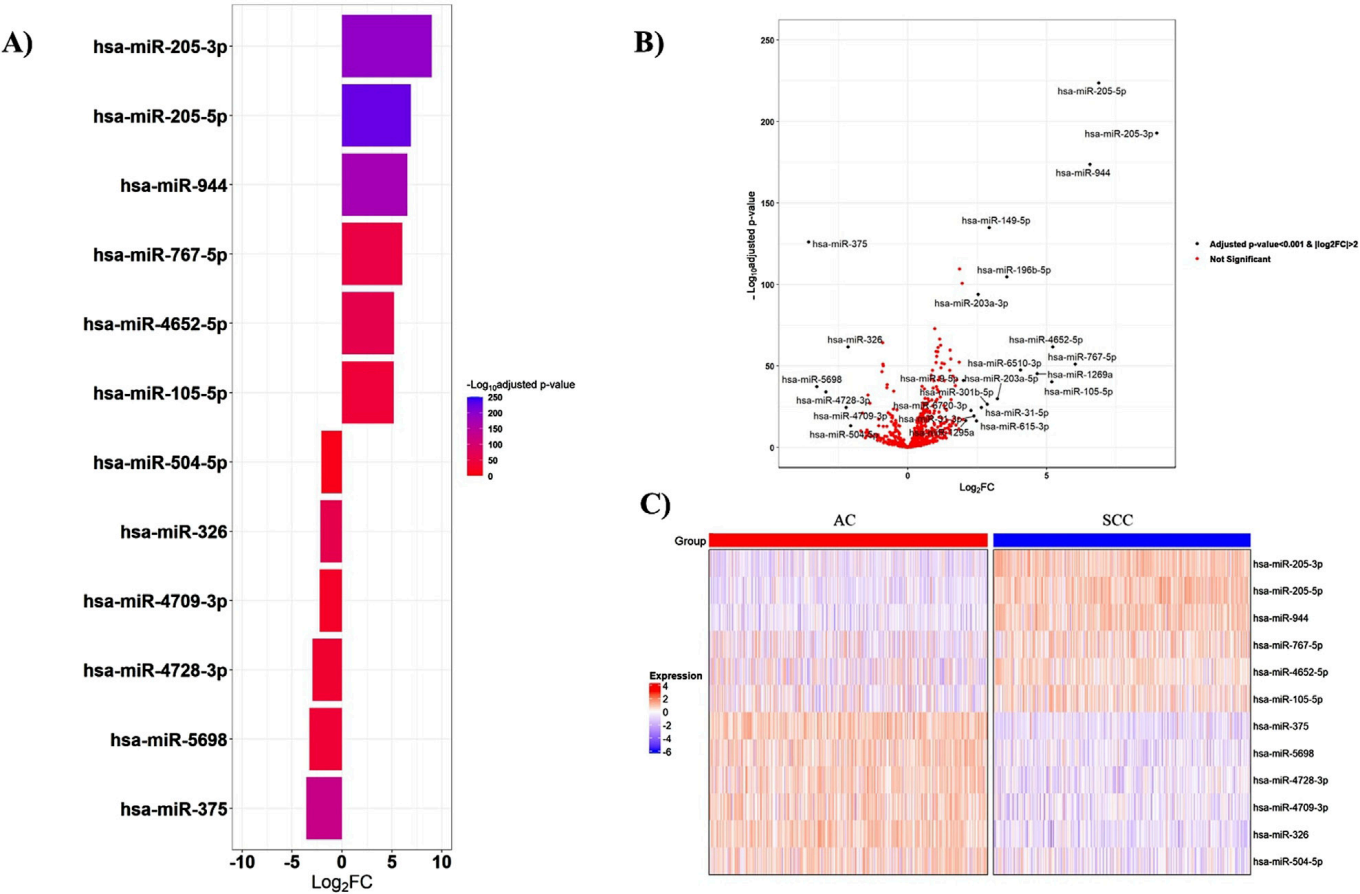
Top-ranked DEMs between AC and SCC samples. **(A)** The bar plot visualizes the top six upregulated and downregulated DEMs, **(B)** The volcano plot represents all significant DEMs with an adjusted *p*-value <0.001 and |log2FC|>2, **(C)** The heatmap shows the expression patterns of miRNAs in 518 AC and 478 SCC samples. The expression levels of miRNAs were log2-transformed. FC: Fold change.

According to the cutoff criteria of |log2FC|>2 and adjusted *p*-value < 0.001, the miRNA mature expression profiles between 518 AC and 478 SCC samples uncovered 25 significantly DEMs, with nineteen upregulated and six downregulated in SCC tissues compared to AC ones. Among these, miR-205-3p, miR-205-5p, miR-944, and miR-767-5p, exhibiting fold change values greater than 6, emerged as the top four DEMs. The miRNAs with the most significant expression differences are presented in Figure 2. Additionally, the expression levels of the top six upregulated and downregulated miRNAs are provided in Supplementary Figures S1–S2.

### 3.2 Identification of significant miRNAs by supervised machine learning approaches

Three different machine learning methods (SVM, Naïve Bayes and Rpart) were employed to determine the potential diagnostic miRNA biomarkers. All methods underwent a 5-fold cross-validation process. The sets of miRNAs selected by each method were almost identical and merged using RRA. The miRNAs with the highest accuracy in discriminating between AC and SCC patients are listed in Table 1.

**TABLE 1:** Ranking of the top 10 miRNAs selected by machine learning methods. SVM: Support Vector Machine. Rpart: Recursive Partitioning and Regression Trees.

miRNA	Aggregate Score	SVM	Rpart	Naïve Bayes
miR-205-3p	7e-12	1	2	2
miR-205-5p	7e-12	3	1	1
miR-944	2.6e-11	2	3	3
miR-6499-3p	6.4e-11	4	4	4
miR-375	1.25e-10	5	5	5
miR-6499-5p	2.16e-10	6	6	6
miR-196b-5p	3.43e-10	7	7	7
miR-326	5.12e-10	8	8	8
miR-149-5p	1.323e-9	11	9	11
miR-6512-3p	1.6e-9	10	12	10

### 3.3 Evaluation of the diagnostic values of selected miRNAs

**FIGURE 3:**
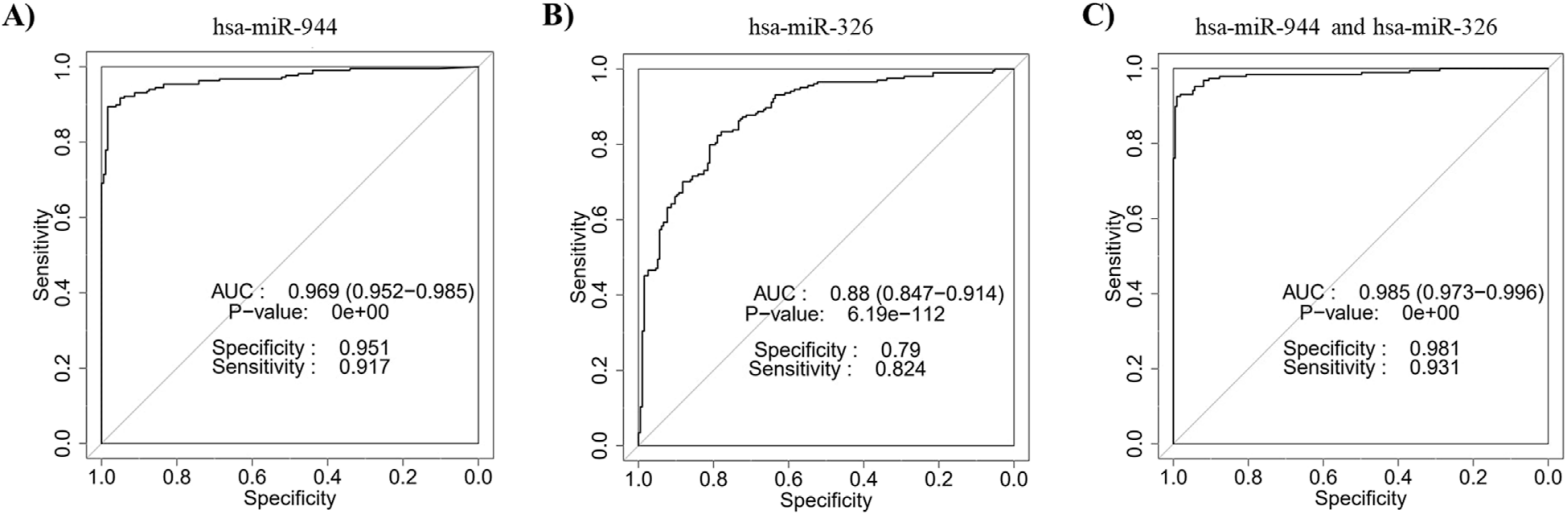
Discriminatory ability of miR-944 and miR-326 for differentiating between AC and SCC based on TCGA data. ROC curve analysis of **(A)** miR-944 demonstrated an AUC of 0.969 with a *p*-value of 0, **(B)** miR-326 showed an AUC of 0.88 with a *p*-value of 6.19e-112, and **(C)** the combination of miR-944 and miR-326 yielded an AUC of 0.985 with a *p*-value of 0, indicating that the integration of the two miRNAs provides higher accuracy for differentiating between AC and SCC tissues compared to either miRNA alone. AUC: Area under the curve.

Five miRNAs (miR-205-3p, miR-205-5p, miR-944, miR-375 and miR-326) were identified as common candidates through both differential expression analysis and feature selection methods. Given the extensively studied expression profiles of miR-205 and miR-375 in NSCLC, we selected miR-944 and miR-326 for experimental validation (Patnaik et al., 2015; Charkiewicz et al., 2016; Lebanony et al., 2009; Del Vescovo et al., 2011; Jin et al., 2015; Chen et al., 2017; Hamamoto et al., 2013). Subsequently, ROC curve analysis was performed to assess the diagnostic value of the combination of these two miRNAs in differentiating between AC and SCC patients. The results revealed that the integration of miR-944 with miR-326 yielded an AUC of 0.985 (95% CI = 0.973-0.996, *p*-value = 0) (Figure 3).

### 3.4 Correlation between miRNA expression and clinicopathological features

To evaluate the relationship between the expression levels of the mentioned miRNAs and clinicopathological characteristics, including sex, age, smoking history, tumor location, pathologic stage, and tumor node metastasis (TNM) stage, correlation analysis was conducted. As indicated in Table 2, among patients with AC, the expression of miR-944 showed significant correlations with smoking history (*p*-value = 0.0098), tumor (T) stage (*p*-value = 0.0162) and lymph node (N) stage (*p*-value = 0.0015). Notably, miR-326 also exhibited correlations with smoking history (*p*-value = 0.0069) and tumor location (*p*-value = 0.0064) in AC patients. In the same cohort, miR-205-5p demonstrated a significant correlation with smoking history (*p*-value = 0.0080), while miR-205-3p was associated with tumor location (*p*-value = 0.0091). For patients diagnosed with SCC, miR-944 expression was significantly associated with sex (*p*-value = 0.0471) and smoking history (*p*-value = 0.0425). However, no significant association was observed with miR-326 in SCC patients. Moreover, both 205-5p and 205-3p were found to be correlated with sex (*p*-value = 0.0079 and *p*-value = 0.0016, respectively) and miR-375 exhibited correlations with tumor location (*p*-value = 0.0263) and N stage (*p*-value = 0.0327) in SCC patients (Table 3).

**TABLE 2:** Correlations between the expression of miRNAs and clinicopathological features in AC. SE: Standard Error.

Variable	Number	Hsa-miR-944		Hsa-miR-326		Hsa-miR-375		Hsa-miR-205-5p		Hsa-miR-205-3p	
		Mean ± SE	*p*-value	Mean ± SE	*p*-value	Mean ± SE	*p*-value	Mean ± SE	*p*-value	Mean ± SE	*p*-value
Gender											
Female	255	21.45 ± 2.80	0.9043	120.32 ± 10.04	0.2293	134,886.47 ± 12,672.73	0.9663	3,216.73 ± 676.05	0.7112	1.52 ± 0.25	0.3839
Male	227	22.04 ± 4.01		103.90 ± 9.23		222,475.52 ± 33,513.81		2,868.74 ± 652.37		1.63 ± 0.35	
Age at Diagnosis											
>60	318	18.94 ± 2.68	0.552	108.20 ± 7.68	0.9281	138,898.64 ± 13,697.27	0.2416	2,728.76 ± 540.90	0.594	1.45 ± 0.26	0.4826
≤60	146	23.81 ± 4.83		109.64 ± 13.48		239,193.89 ± 45,960.59		2,601.64 ± 475.79		1.43 ± 0.25	
Tobacco Smoking History											
Lifelong Non-smoker	70	28.31 ± 5.97	0.0098	133.14 ± 15.23	0.0069	120,550.61 ± 14,524.92	0.7861	2,938.63 ± 702.88	0.0080	1.7 ± 0.40	0.0746
Current smoker	108	20.18 ± 6.08		107.60 ± 14.00		260,183.41 ± 53,669.74		3,687.90 ± 1,290.05		1.74 ± 0.47	
Former smoker	292	20.23 ± 2.90		108.63 ± 9.14		156,934.16 ± 19,653.48		2,899.63 ± 591.16		1.48 ± 0.28	
Tumor Location											
Central Lung	60	12.27 ± 2.59	0.1196	77.48 ± 12.73	0.0064	272,808.8 ± 80,673.95	0.45	1,018.15 ± 217.29	0.1863	0.45 ± 0.12	0.0091
Peripheral Lung	118	28.69 ± 6.08		115.97 ± 14.16		206,453.74 ± 43,352.85		4,682.40 ± 1,480.30		2.54 ± 0.68	
Pathologic Stage											
Stage I	260	20.58 ± 3.00	0.2424	116.6 ± 9.95	0.6213	161,574.37 ± 19,370.76	0.9872	3,129.89 ± 547.42	0.3765	1.43 ± 0.22	0.6648
Stage II	116	17.35 ± 2.84		120.23 ± 14.96		155,022.51 ± 26,174.17		2,205.53 ± 488.15		1.43 ± 0.30	
Stage III	76	34.53 ± 9.89		97.13 ± 13.20		145,879.91 ± 28,638.29		4,910.34 ± 2,196.40		2.66 ± 1.01	
Stage IV	23	19.22 ± 9.54		96.96 ± 20.76		487,458.78 ± 217,966.33		1,160 ± 468.93		0.78 ± 0.40	
T Stage											
T1+2	417	23.95 ± 2.75	0.0162	111.91 ± 7.11	0.6101	179,460.99 ± 19,237.40	0.7639	3,232 ± 537.89	0.3032	1.63 ± 0.24	0.2815
T3+4	62	7.65 ± 1.29		118.55 ± 23.93		161,061.18 ± 35,099.26		1885.06 ± 547.05		1.23 ± 0.41	
N Stage											
No	313	18.72 ± 2.58	0.0015	118.50 ± 9.38	0.1771	175,982.89 ± 19,719.05	0.8135	2,875.93 ± 467.48	0.5801	1.37 ± 0.19	0.4777
Yes	158	27.44 ± 5.17		100.54 ± 9.39		180,559.69 ± 35,251.41		3,500.75 ± 1,099.53		2 ± 0.51	
M Stage											
No	327	20.18 ± 2.88	0.6466	109.82 ± 9.14	0.9365	162,321.87 ± 18,184.31	0.6412	2,755.57 ± 571.67	0.5111	1.51 ± 0.27	0.44
Yes	22	19.82 ± 9.97		99.32 ± 21.59		509,246.23 ± 226,967.22		1,212.59 ± 487.66		0.82 ± 0.41	

**TABLE 3:** Correlations between the expression of miRNAs and clinicopathological features in SCC. SE: Standard Error.

Variable	Number	hsa-miR-944		hsa-miR-326		hsa-miR-375		hsa-miR-205-5p		hsa-miR-205-3p	
		Mean ± SE	*p*-value	Mean ± SE	*p*-value	Mean ± SE	*p*-value	Mean ± SE	*p*-value	Mean ± SE	*p*-value
Gender											
Female	110	502.47 ± 50.23	0.0471	23.67 ± 2.95	0.9996	40,701.25 ± 1,6246.90	0.095	42,425.55 ± 3,759.23	0.0079	18.96 ± 1.71	0.0016
Male	332	634.02 ± 42.73		23.67 ± 2.36		12,742.31 ± 3,468.48		54,673.06 ± 2,602.88		25.65 ± 1.20	
Age at Diagnosis											
>60	340	586.86 ± 40.36	0.1457	23.09 ± 2.11	0.8979	21,378.16 ± 6,138.90	0.3427	51,802.44 ± 2,479.60	0.8293	23.86 ± 1.15	0.8899
≤60	94	673.48 ± 70.14		22.13 ± 4.07		13,992.39 ± 4,769.67		51,152.89 ± 4,735.70		24.62 ± 2.19	
Tobacco Smoking History											
Lifelong Non-smoker	16	614 ± 268.75	0.0425	21.19 ± 5.60	0.0593	16,579.69 ± 6,435.33	0.3612	41,660.56 ± 10,087.78	0.1861	21 ± 4.33	0.3253
Current smoker	126	660.29 ± 60.42		24.93 ± 3.07		14,238.14 ± 5,328.52		55,049.92 ± 3,997.03		24.96 ± 1.71	
Former smoker	290	571.67 ± 42.86		22.87 ± 2.55		22,379.75 ± 6,978.98		50,937.22 ± 2,766.96		23.90 ± 1.31	
Tumor Location											
Central Lung	127	680.09 ± 62.55	0.9372	19.12 ± 2.45	0.1163	8,513.74 ± 1,192.26	0.0263	58,150.56 ± 3,722.64	0.1784	27.23 ± 1.86	0.3871
Peripheral Lung	85	649.69 ± 66.42		34.09 ± 5.79		38,956.54 ± 15,679.78		55,968.73 ± 5,750.01		25.22 ± 2.29	
Pathologic Stage											
Stage I	211	548.81 ± 36.78	0.1079	22.32 ± 1.88	0.2779	19,241.17 ± 7,661.68	0.0625	50,768.91 ± 2,914.26	0.2684	24.47 ± 1.47	0.0667
Stage II	150	683.25 ± 65.80		25.49 ± 3.75		14,116.67 ± 4,716.49		56,421.73 ± 4,143.13		26.2 ± 1.83	
Stage III	72	620.79 ± 118.78		25.35 ± 6.83		34,525.79 ± 16,727.80		46,885.43 ± 5,451.30		18.72 ± 2.11	
Stage IV	6	218.67 ± 85.39		13.5 ± 9.61		6,754.33 ± 3,051.98		34,465.33 ± 11,423.46		20.83 ± 7.35	
T Stage											
T1+2	360	587.83 ± 33.83	0.3963	23.69 ± 2.06	0.8502	19,771.54 ± 5,760.32	0.4458	51,407.13 ± 2,341.05	0.569	24.42 ± 1.12	0.2639
T3+4	82	660.34 ± 112.49		23.61 ± 5.03		19,388.15 ± 6,294.61		52,581.73 ± 5,728.39		22.10 ± 2.29	
N Stage											
No	279	615.97 ± 42.05	0.3133	22.94 ± 2.12	0.368	17,531.96 ± 5,853.17	0.0327	53,823.54 ± 2,850.50	0.2108	25.02 ± 1.31	0.1952
Yes	157	582.24 ± 62.02		24.11 ± 3.81		23,486.78 ± 8,775.43		48,627.02 ± 3,431.85		22.45 ± 1.60	
M Stage											
No	361	565.144 ± 33.90	0.0832	20.66 ± 1.75	0.1698	16,718.19 ± 3,929.55	0.9243	51,018.11 ± 2,468.71	0.4185	23.90 ± 1.13	0.7859
Yes	6	218.67 ± 85.39		13.5 ± 9.61		6,754.33 ± 3,051.98		34,465.33 ± 11,423.46		20.83 ± 7.35	

### 3.5 Evaluation of the prognostic values of selected miRNAs

**FIGURE 4:**
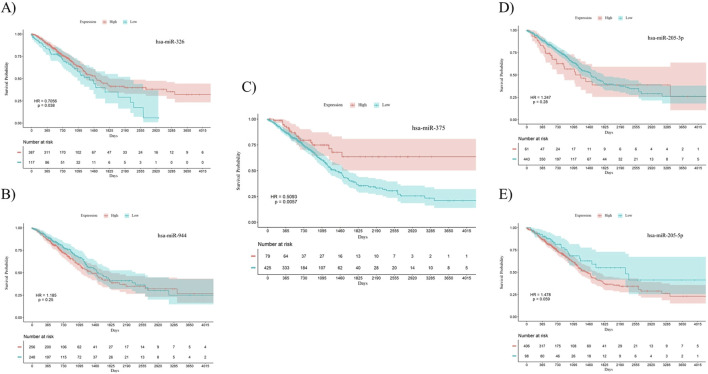
Survival analysis using Kaplan-Meier curves in patients with AC. (A) miR-326, **(B)** miR-944, **(C)** miR-375, **(D)** miR-205-3p, **(E)** miR-205-5p. miR-326 (*p*-value = 0.038) and miR-375 (*p*-value = 0.0057) were significantly correlated with overall survival in AC patients. The log-rank test was used to determine the *p*-values for the survival analysis of these miRNAs. HR: Hazard Ratio, p: *p*-value.

**FIGURE 5:**
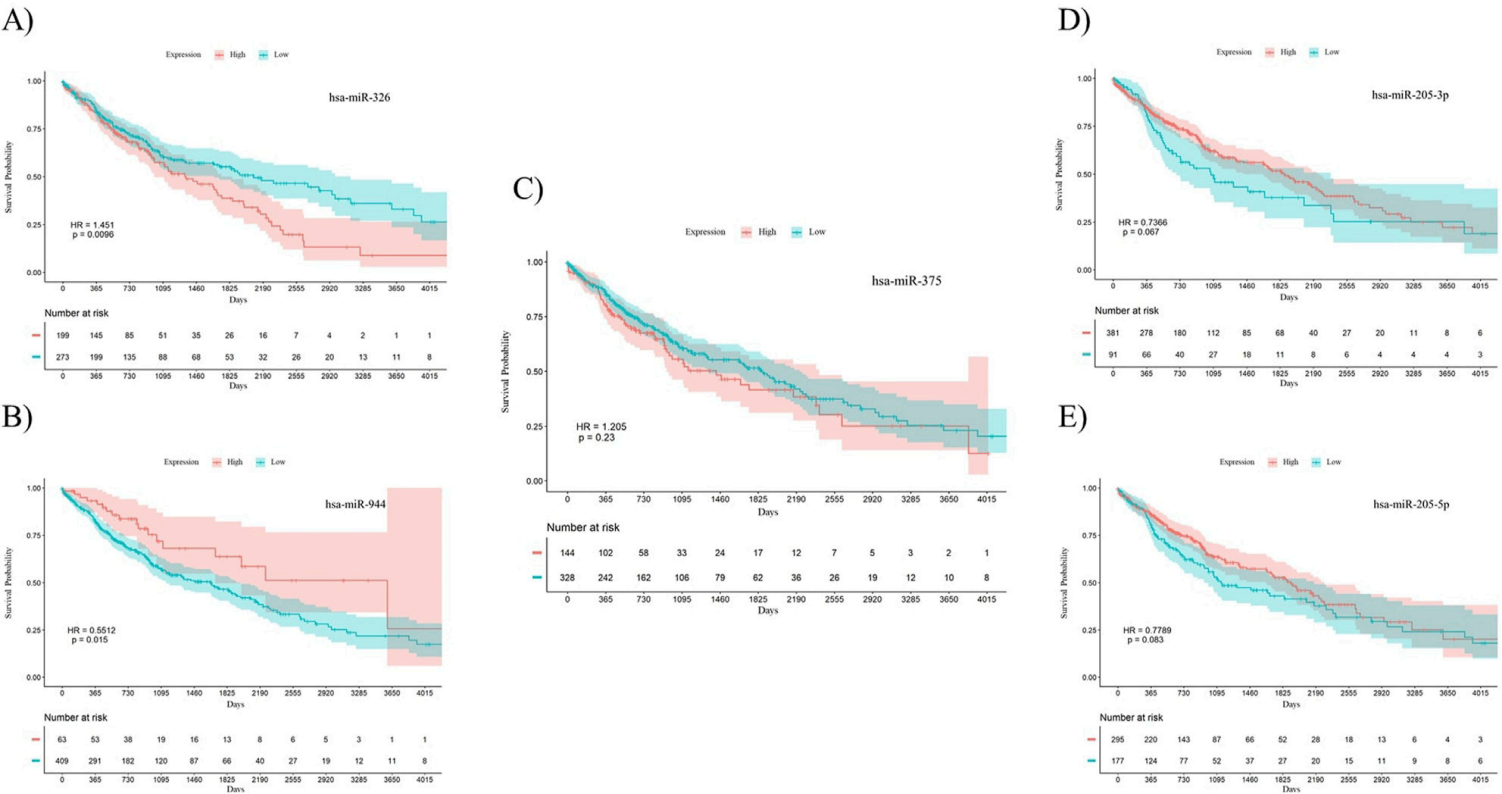
Survival analysis using Kaplan-Meier curves in patients with SCC. (A) miR-326, **(B)** miR-944, **(C)** miR-375, **(D)** miR-205-3p, **(E)** miR-205-5p. miR-326 (*p*-value = 0.0096) and miR-944 (*p*-value = 0.015) were significantly correlated with overall survival in SCC patients. The log-rank test was used to determine the *p*-values for the survival analysis of these miRNAs. HR: Hazard Ratio, p: *p*-value.

To investigate the prognostic power of the five miRNAs identified through both differential expression analysis and feature selection methods, separate Kaplan-Meier analyses were conducted for both AC and SCC subtypes. This involved dividing samples into two groups based on high and low levels of miRNA expression. The results indicate that miR-326 (Hazard Ratio (HR) = 0.7056, *p*-value = 0.038) and miR-375 (HR = 0.5093, *p*-value = 0.0057) demonstrated statistically significant associations with the overall survival of patients with AC. Meanwhile, miR-326 (HR = 1.451, *p*-value = 0.0096) and miR-944 (HR = 0.5512, *p*-value = 0.015) showed associations with the overall survival of patients diagnosed with SCC. The overall survival plots for the five mentioned miRNAs in AC are presented in Figure 4, while the corresponding plots for SCC are shown in Figure 5.

### 3.6 RT-qPCR validation of the candidate miRNAs

**FIGURE 6:**
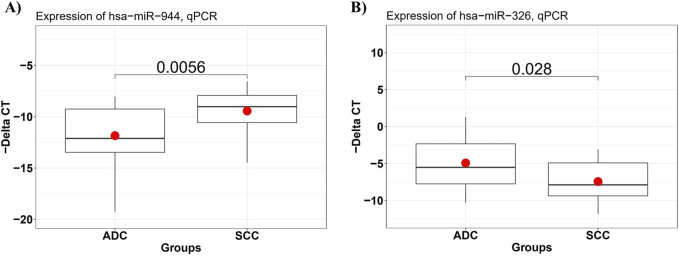
The expression level of miRNAs in AC and SCC tissue samples detected by RT-qPCR. **(A)** miR-944 expression levels were significantly increased in SCC compared to AC samples, with a *p*-value of 0.0056, and **(B)** miR-326 expression levels were significantly lower in SCC tissues, with a *p*-value of 0.028.

To confirm the differential expression of miR-944 and miR-326 in SCC samples compared to AC tissues, RT-qPCR was performed to assess the relative expression of these miRNAs in 29 AC and 21 SCC FFPE tissue specimens. The results of RT-qPCR were consistent with the findings from our bioinformatic analysis. We observed that, in comparison to AC samples, the relative expression level of miR-944 was significantly higher in SCC specimens (*p*-value = 0.0056), whereas miR-326 exhibited a lower expression level in SCC compared to AC tissues (*p*-value = 0.028) (Figure 6).

### 3.7 Diagnostic value of the candidate miRNAs in distinguishing between SCC and AC based on RT-qPCR

**FIGURE 7:**
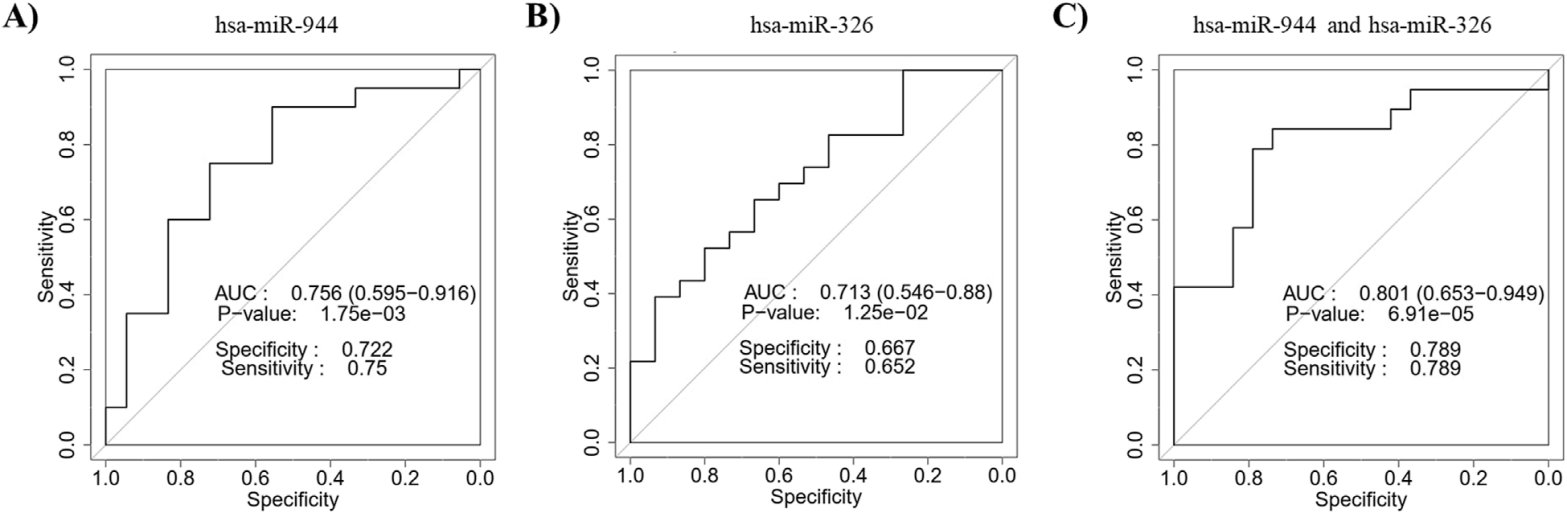
Discriminatory ability of miR-944 and miR-326 for differentiating between AC and SCC based on RT-qPCR data. ROC curve analysis of **(A)** miR-944 demonstrated an AUC of 0.756 with a *p*-value of 1.75e-03, **(B)** miR-326 showed an AUC of 0.713 with a *p*-value of 1.25e-02, and **(C)** the combination of miR-944 and miR-326 yielded an AUC of 0.801 with a *p*-value of 6.91e-05. These findings confirm the bioinformatics analysis, indicating that the combined use of these miRNAs improves distinguishing power. AUC: Area under the curve.

ROC curves based on the RT-qPCR data were constructed to evaluate the diagnostic power of miR-944 and miR-326 for differentiating between AC and SCC. The ROC curve of miR-944 showed an AUC of 0.756, with a sensitivity of 75% and specificity of 72.2%, while miR-326 exhibited an AUC value of 0.713, with a sensitivity of 65.2% and specificity of 66.7%. In addition, the combination of the two miRNAs obtained an AUC of 0.801 with a sensitivity of 78.9% and specificity of 78.9%, indicating an improvement in diagnostic performance (Figure 7).

### 3.8 Target genes and functional enrichment analysis

To enhance the reliability of subsequent analyses, we investigated the target genes of the top 25 DEMs using miRTarBase, a database renowned for providing experimentally validated miRNA-target interactions (Huang et al., 2022). We specifically limited the selection of target genes to those supported by strong experimental evidence, resulting in the identification of 483 genes. Following this, we assessed the negative correlation between these miRNAs and their target genes by employing NSCLC samples obtained from TCGA, revealing 130 genes with a significant negative correlation. Target genes with a correlation *p*-value <0.05 were then utilized as input for KEGG pathway and GO analyses.

**FIGURE 8:**
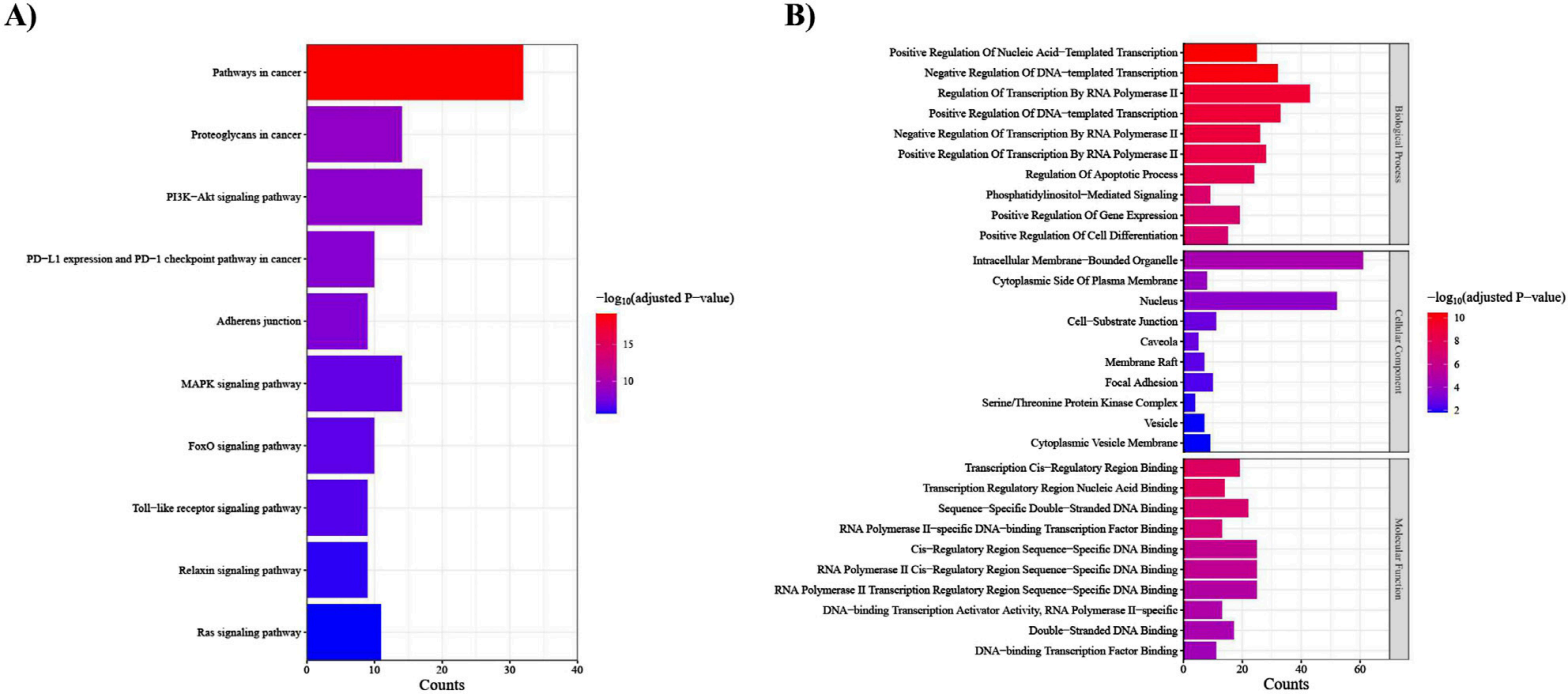
Functional enrichment analysis of the top 25 DEMs target genes. (A) KEGG pathways; the *x*-axis indicates the number of target genes involved in each pathway, which is shown on the *y*-axis, **(B)** Gene ontology (GO) analysis; the *x*-axis indicates the number of target genes, and the *y*-axis represents the GO terms, including Biological Process, Cellular Component, and Molecular Function.

The KEGG pathway analysis uncovered that our target genes are primarily involved in pathways in cancer, proteoglycans in cancer, PI3K-Akt signaling pathway, and PD-L1 expression and PD-1 checkpoint pathway in cancer (Figure 8A). In the context of GO enrichment analysis, Figure 8B illustrates the top 10 associated terms in biological processes (BP), cellular components (CC), and molecular functions (MF).

## 4 Discussion

In this study, we retrieved mature miRNA expression profiles along with the relevant clinical information of AC and SCC patients from TCGA database. Employing the “Limma” R package, we conducted differential expression analysis to identify miRNAs that display notable variations in expression levels between AC and SCC. Our findings unveiled a significant upregulation in the expression levels of miR-205-3p, miR-205-5p, miR-944, miR-767-5p, miR-4652-5p, and miR-105-5p, contrasting with a marked downregulation in the levels of miR-504-5p, miR-326, miR-4709-3p, miR-4728-3p, miR-5698, and miR-375 observed in SCC patients compared to individuals diagnosed with AC. We subsequently assessed the diagnostic accuracy of miRNAs through the application of three different supervised machine learning approaches: SVM, Decision Trees and Naïve Bayes. Among the top 10 ranked miRNAs with the utmost accuracy for discriminating between AC and SCC, five miRNAs were found to overlap with the highest-ranked DEMs: miR-205-3p, miR-205-5p, miR-944, miR-375 and miR-326.

Further clinical importance of the identified miRNAs was elucidated through the assessment of their prognostic potential, employing TCGA samples. In patients with AC, elevated expression levels of miR-375 and miR-326 were found to be significantly correlated with an increased overall survival rate. Furthermore, the relationship between miR-326 expression level and overall survival exhibited a reversal among SCC patients, where higher expression correlated with a shorter overall survival. Additionally, SCC patients presenting low miR-944 expression levels experienced a significantly poorer overall survival outcome compared to their counterparts with high expression levels.

All five of miRNAs identified in our study have been reported to be involved in NSCLC tumorigenesis. In line with our study, Ma et al. demonstrated a significant increase in the expression level of miR-944 within SCC tumors. Furthermore, they confirmed that the upregulated expression of this miRNA stimulated cell growth, proliferation, migration, and invasion. Functioning as an oncogene, miR-944 targets SOCS4, a tumor suppressor gene involved in the regulation of JAK/STAT signaling pathway (Ma et al., 2014).In contrast, An et al. revealed the tumor-suppressive role of miR-944 and its participation in the JAK/STAT signaling pathway by directly targeting STAT1. They also observed a reduction in the expression of this miRNA in AC tissues and cell lines (An et al., 2019). Other studies provided additional evidence supporting the suppressor role of miR-944 in the proliferation, invasion, and migration in NSCLC cell lines through its binding to MACC1, EPHA7, YES1, ETS1, and LASP1 (Zhu et al., 2022; Liu et al., 2016; Lv et al., 2021; Shen et al., 2022).

Regarding miR-326, its tumor-suppressive activity has been revealed in various studies. Overexpression of miR-326 represses the proliferation, invasion, and migration in NSCLC cells. This inhibitory effect is achieved by targeting different genes implicated in the progression of LC, including CCND1, NSBP1, Phox2a, and CD155 (Sun et al., 2016; Wang et al., 2016; Nakanishi et al., 2023; Li et al., 2016). Literature presents conflicting data on the expression level of miR-326 in AC. While certain studies indicate its attenuation, our results align with others showing an upregulation of miR-326 in AC samples (Sun et al., 2016; Charkiewicz et al., 2023). Moreover, consistent with our findings, Gan et al. revealed its downregulation in SCC along with a negative correlation with survival, reinforcing our study’s results (Gan et al., 2019).

To the best of our knowledge, there have been limited studies on the mechanism of miR-375 in NSCLC, necessitating further investigation. Nevertheless, the deregulation of this miRNA and its association with survival in NSCLC have been evaluated in prior studies. Our findings are consistent with existing reports, supporting the downregulation of miR-375 in SCC and its upregulation in AC. Furthermore, its low expression correlates with shorter overall survival, suggesting a potential tumor suppressor role in AC (Jin et al., 2015; Li et al., 2012; Chen et al., 2017).

Despite inconsistencies in the available data regarding the role of miR-205 in LC, the majority of studies indicate its tumor-promoting function. It is involved in stimulating cell proliferation, invasion, and migration in NSCLC by targeting genes with tumor-suppressive roles, such as PTEN, PHLPP2, SMAD4, and TP53INP1 (Cai et al., 2013; Zhao et al., 2022; Zeng et al., 2017; Lei et al., 2013). The expression levels of this miRNA have been evaluated not only in NSCLC compared to adjacent normal tissues but also separately in AC and SCC. Consequently, elevated miR-205 expression was observed in NSCLC samples, with higher levels detected in the SCC subtype compared to AC tissues (Duan et al., 2017; Xu et al., 2021). This observation suggests the potential of miR-205 as a discriminatory marker for distinguishing between SCC and AC, supported by our study and confirmed by Lebanony et al. (Lebanony et al., 2009).

These findings suggest that similar to the expression patterns of the five miRNAs specific to the NSCLC subtype, their functional roles may vary depending on whether it is AC or SCC. Our study provides further support for this observation, particularly through the correlation analysis between miRNAs and overall survival. For instance, miR-326 demonstrated a completely opposite correlation within AC samples compared to SCC, indicating potential divergent roles in these two subtypes. This observation underscores the necessity for experimental validation to elucidate their possible distinct functions. Hence, we propose an investigation into the exact roles of these miRNAs in NSCLC subtypes separately.

The results of the diagnostic power evaluation revealed that the combination of miR-944 and miR-326 achieved an AUC value of 0.985, demonstrating a sensitivity of 0.932 and specificity of 0.981. It is essential to note that our RT-qPCR results did not match the robustness of the bioinformatics analysis, yielding an AUC of 0.801. This discrepancy can be attributed, in part, to limitations in our sample size. We hypothesize that with a larger sample cohort, our experimental results would align more closely with the bioinformatics outcomes.

Moreover, correlation analysis indicated a significant association between the expression levels of miR-944, miR-326, and miR-205-5p and smoking history in individuals with AC. Considering that AC represents the most prevalent subtype among nonsmokers, numerous studies have investigated molecular alterations, revealing distinctions not only at the genetic level but also in expression profiles between never-smokers and ever-smokers (Zhang et al., 2018; Li et al., 2018; Inamura et al., 2016). Our findings suggest that differences in miRNA expression patterns are also evident between the two specified groups.

Our functional enrichment analysis revealed that the top 25 DEMs identified in our study exert pivotal influences on key pathways contributing to the development and progression of NSCLC (Yuan et al., 2019; Mosele et al., 2020). These pathways include PI3K-Akt, MAPK, FoxO, Ras signaling pathways, as well as PD-L1 expression and PD-1 checkpoint pathway. Currently, numerous drugs are available, targeting the genes regulated by these miRNAs. For instance, PIK3CA, PDGFRB, VEGFA, and IGF1R emerge as crucial options for targeted treatment in NSCLC (Hirsch et al., 2017; Lemjabbar-alaoui et al., 2015). Hence, the identified miRNAs may participate in responses to these targeted therapies. It is strongly recommended to evaluate the impact of these drugs on miRNA expression levels. In addition, these miRNAs themselves may play a therapeutic role by regulating the aforementioned pathways. Therefore, exploring their therapeutic potential presents a valuable opportunity for the development of innovative and effective therapies for NSCLC patients.

Traditional histopathological diagnosis, even when combined with immunohistochemical staining, faces several challenges and limitations. These include the difficulty of diagnosing small biopsy samples from patients with unresectable NSCLC, cases of NSCLC not otherwise specified, and discrepancies in diagnoses among pathologists (Lindquist et al., 2022; Yamashita et al., 2023). Therefore, the development of a differentiating approach that does not rely on conventional methods is crucial for enhancing diagnostic precision. Detecting miRNAs as discriminative markers using RT-qPCR offers numerous advantages. MiRNAs remain stable in FFPE tissues due to their short length, and RT-qPCR, being a quantitative method, eliminates the need for expertise in interpretation (Sinn et al., 2017; Wilkerson et al., 2013). In addition, miR-944, located in the *TP63* gene which is frequently amplified in SCC, can serve as an alternative or complementary biomarker to p40, an isoform of the p63 protein used as an IHC marker for SCC (Rodriguez-canales et al., 2016).

Numerous previous studies have aimed to detect miRNAs with high precision to differentiate between AC and SCC. Early research focused on identifying a single miRNA as a distinguishing biomarker, leading to the identification of miR-205. miR-205 was initially considered a stand-alone biomarker with sufficient sensitivity and specificity to independently differentiate between the two subtypes (Lebanony et al., 2009; Bishop et al., 2010). However, subsequent studies revealed that miR-205 alone was less effective than previously believed and should be combined with another miRNA to enhance accuracy (Del Vescovo et al., 2011). This resulted in the identification of miR-375 as a promising candidate to pair with miR-205 (Patnaik et al., 2015; Hamamoto et al., 2013). Despite this progress, there remained potential for improving the sensitivity and specificity by adding more miRNAs to this panel. Hence, we conducted comprehensive analyses beyond simple differential expression, employing machine learning algorithms such as SVM, Naïve Bayes, and Rpart, to enhance accuracy. The purpose of this study was to: first, determine if miR-205 and miR-375 are indeed among the top-ranked miRNAs for differentiating AC and SCC in the large TCGA cohort; and second, identify other potentially valuable miRNAs that can be combined with miR-205 and miR-375 to form a panel with high sensitivity and specificity.

## 5 Conclusion

Our study aimed to address the imperative need for accurate differentiation between AC and SCC through the exploration of miRNAs. Utilizing bioinformatics analyses, we not only identified miRNAs with significant discriminatory capabilities in distinguishing between AC and SCC but also provided valuable insights into the potential roles of miRNAs as prognostic factors. We demonstrated their correlation with clinicopathological features in these specific cancer subtypes. Furthermore, by predicting the target genes of miRNAs and conducting thorough pathway and GO enrichment analyses, this study sought to unveil the functional mechanisms of the top-ranked miRNAs. Ultimately, our study establishes a foundation for future research and underscores the clinical significance of miRNA-based stratification in NSCLC, paving the way for more precise diagnoses and, consequently, more personalized and targeted therapeutic interventions.

## Data Availability

The original contributions presented in the study are included in the article/Supplementary Material, further inquiries can be directed to the corresponding author.
