# Supplementary material for: A comprehensive in silico analysis and experimental validation of miRNAs capable of discriminating between lung adenocarcinoma and squamous cell carcinoma
Source: Front Genet. 2024 Sep 23;15:1419099. doi: 10.3389/fgene.2024.1419099 (PMC11460580; doi:10.3389/fgene.2024.1419099)
Supplement: Supplementary file 1 [file Table1.DOCX]

| **Gene** | **Primer** | **Band Size** | **Sequence (5´- 3´)** |
| --- | --- | --- | --- |
| hsa-miR-944 | Forward | 78 | CGCCAAATTATTGTACATCGGATG |
| hsa-miR-326 | Forward | 73 | CGCCTCTGGGCCCTTCCTCC |
| SNORD48 | Forward | 106 | TGACCCCAGGTAACTCTGAGTGTGT |
|  | Universal Reverse Primer |  | CCAGTGAGCAGAGTGACG |
|  | Anchored Oligo dT mix |  | CCAGTGAGCAGAGTGACGAGGACTCGAGCTCAAGCTTTTTTTTTTTTTTTT(V) |

**Supplementary Table 1. Sequence of qRT-PCR primers.**
