# Supplementary material for: A comprehensive in silico analysis and experimental validation of miRNAs capable of discriminating between lung adenocarcinoma and squamous cell carcinoma
Source: Front Genet. 2024 Sep 23;15:1419099. doi: 10.3389/fgene.2024.1419099 (PMC11460580; doi:10.3389/fgene.2024.1419099)
Supplement: Supplementary file 2 [file Table2.docx]

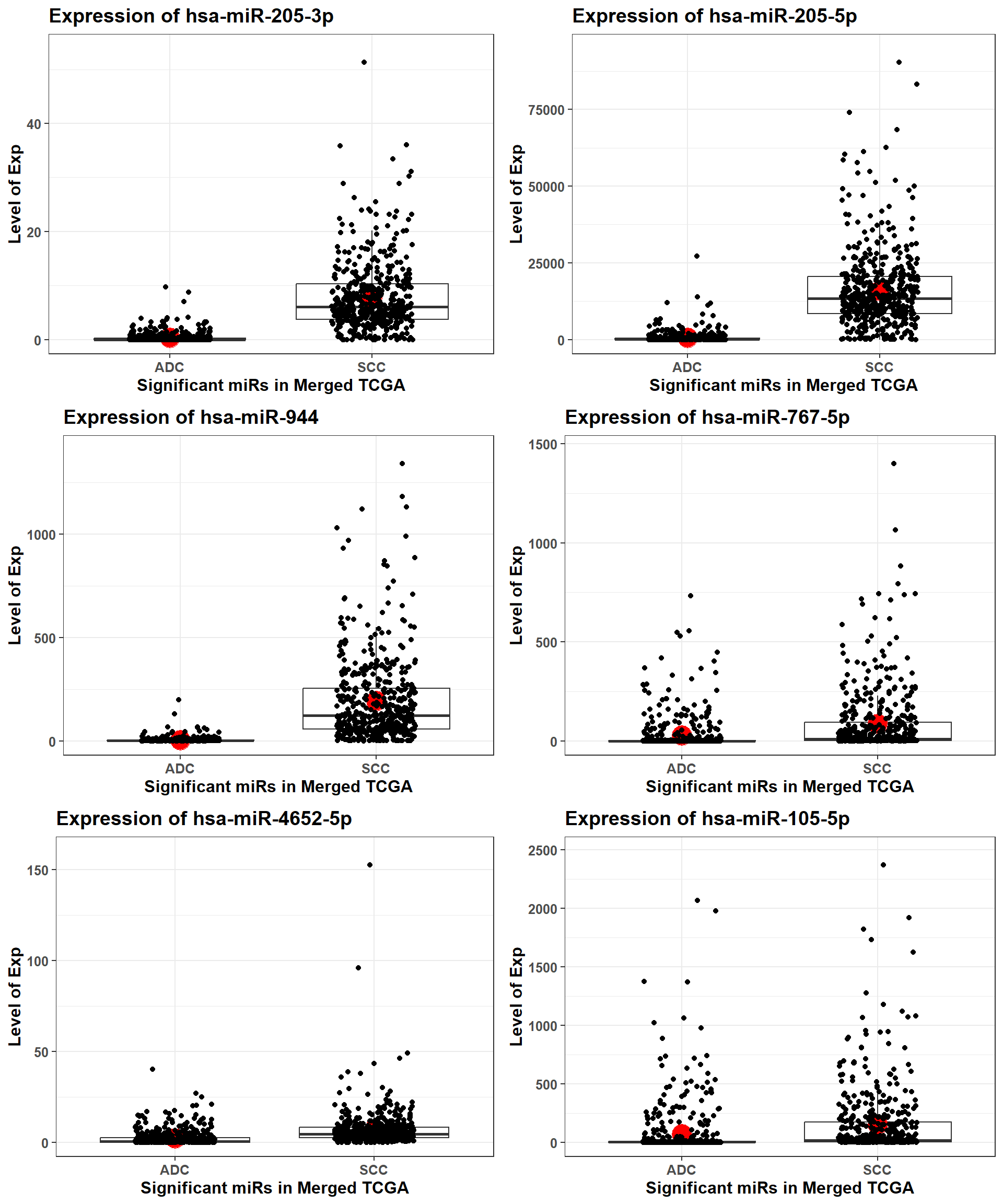
**Supplementary Figure 1.** Comparison of the expression levels of the top 6 upregulated miRNAs in SCC versus AC.


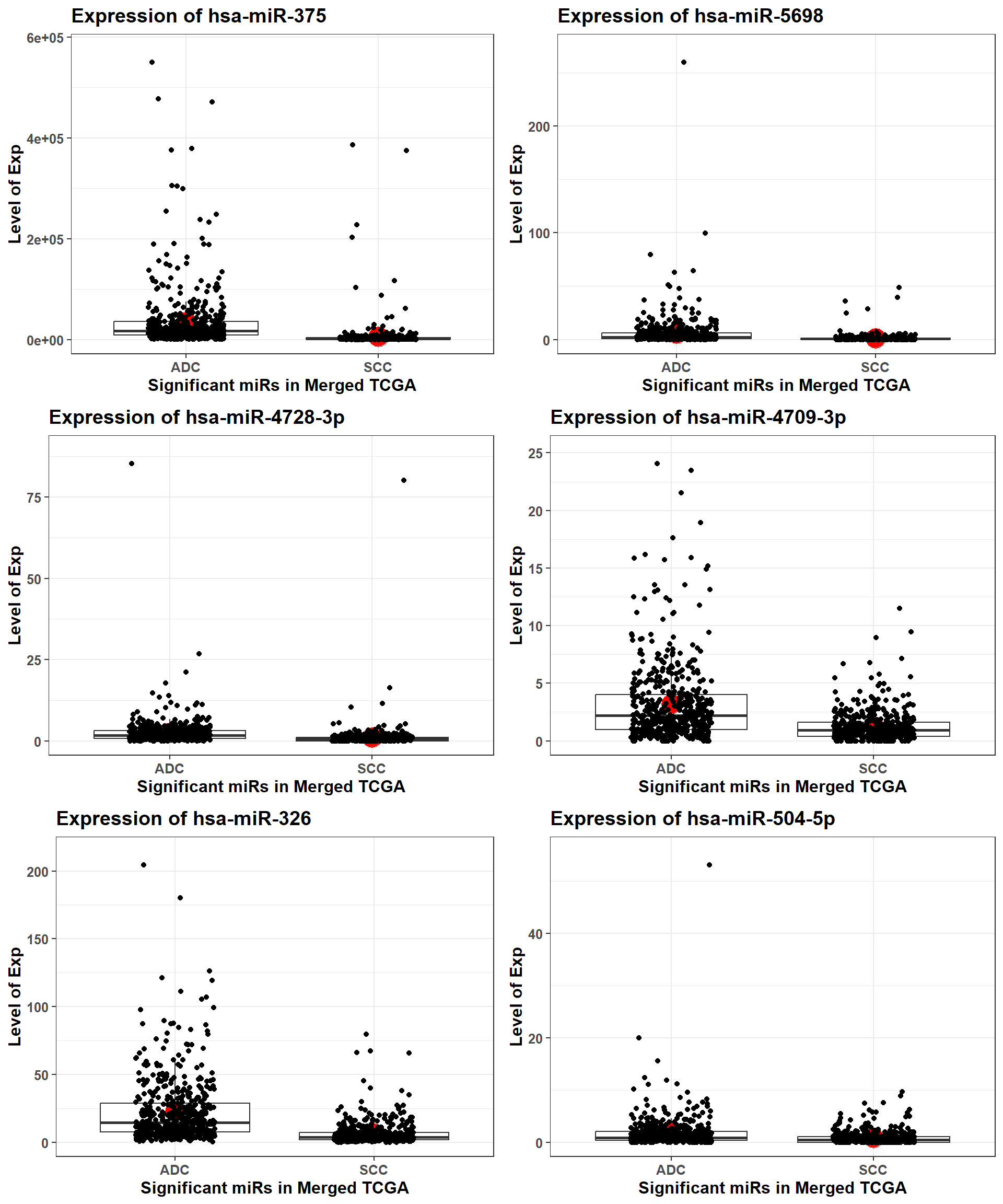
**Supplementary Figure 2.** Comparison of the expression levels of the top 6 downregulated miRNAs in SCC versus AC.
